# Toxicity of *Metarhizium flavoviride* conidia virulence against *Spodoptera litura* (Lepidoptera: Noctuidae) and its impact on physiological and biochemical activities

**DOI:** 10.1038/s41598-022-20426-x

**Published:** 2022-10-06

**Authors:** Perumal Vivekanandhan, Kannan Swathy, Lucy Alford, Sarayut Pittarate, Subramanian Panchu Ravindra Rajan Subala, Supamit Mekchay, Dilipan Elangovan, Patcharin Krutmuang

**Affiliations:** 1 Department of Physiology, Saveetha Dental College and Hospitals, Saveetha Institute of Medical & Technical Sciences, Saveetha University, Chennai, Tamil Nadu 600077 India; 2 Department of Entomology and Plant Pathology, Faculty of Agriculture, Chiang Mai University, Chiang Mai, 50200 Thailand; 3 School of Biological Sciences, Life Sciences Building, University of Bristol, 24 Tyndall Avenue, Bristol, BS8 1TQ United Kingdom; 4 Department of Biotechnology, Periyar University, Salem, Tamil Nadu 636011 India; 5 Department of Animal and Aquatic Sciences, Faculty of Agriculture, Chiang Mai University, Chiang Mai, 50200 Thailand; 6 Innovative Agriculture Research Center, Faculty of Agriculture, Chiang Mai University, Chiang Mai, 50200 Thailand

**Keywords:** Zoology, Entomology

## Abstract

Insect pests of agricultural crops have establish immunological tolerance against fungal infection caused by pathogens via different humoral and cellular processes. Fungal infection can be prevented by insect antioxidant and detoxifying enzymes, but there is no clear understanding of how they physiologically and biochemically interact. Our study aims to examine the antioxidant and detoxifying enzyme defense systems of the pest insect *Spodoptera litura* in response to infection by *Metarhizium flavoviride*. At 48 h following exposure to *M. flavoviride*, antioxidant enzyme levels were modified, and phenoloxidase and total hemocyte count were decreased significantly. The amount of detoxifying enzymes increased significantly. *M. flavoviride* appears to directly affect the *S. litura* immune system and results in decreased immunity. In a bioassay, *M. flavoviride* was found to be harmful to *S. litura* larvae in their third and fourth instar stage. *M. flavoviride* may be an effective tool in the control of *S. litura* larvae. Such entomopathogenic fungi represent cheaper, pollution free, target specific, promising alternatives to synthetic chemical tools in the for control insect pests.

## Introduction

An overuse and overdependence on chemical insecticides in the control of insect pests has led to the development of physiological resistance in pest insects, as well as causing detrimental impacts on beneficial insects such as natural enemies that play key roles in the natural management of pest insect populations. The present-day ineffectiveness of synthetic chemicals in the control of insect pests has led to the widespread development of a variety of biological pesticide alternatives^1–4^, with integrative pest management (IPM) increasingly incorporating the use of insect pathogens into programs^5–8^. Insect pathogens encompass species of viruses, fungi, bacteria and nematodes that are capable of infecting and killing an insect pest in the natural environment^9^. In pursuit of novel and environmentally friendly insect control methods, the study and documentation of fungi has led to the development of several entomopathogenic fungi-based microbial agents for commercial use in the control of insect pests^10,11^. Additional research into the mode of action of entomopathogenic fungi infection is necessary in order to determine the possibility of manipulating fungal virulence factors to increase fungal infection and death^12^.

Almost all microbes that insects encounter in different habitats are controlled by their immune systems. Humoral and cellular defenses make up the innate immune system of insects^13^. Several hemocyte-mediated responses are involved in the protection against fungi infection, including phagocytosis, nodulation, and encapsulation^14^. As such, the morphological and functional characteristics of multiple types of hemocytes have been determined in several orders of insect species^15^. For example, as larvae of lepidoptera undergo their metamorphosis, only granulocytes and plasmatocytes adhere to foreign surfaces^16^, with granulocytes and plasmatocytes commonly representing more than 50% of all hemocytes.

Similar to other eukaryotes, insects have developed a suite of antioxidant enzymes to reduce lipid peroxidation, protein oxidation, and DNA damage due to Reactive oxygen species (ROS)^17^. Superoxide dismutase (SOD), catalase (CAT), and peroxidases (POX) are the main components of insects' antioxidant enzyme systems. As SOD scavenges ROS by breaking down superoxide into oxygen and hydrogen peroxide, it releases oxygen into the environment. In response, CAT and a variety of POX scavenge hydrogen peroxide and produce oxygen and water. A number of enzymes can play an important role in enzymatic defense, including SOD, ACP (Acid Phosphatase), ALP (Alkaline Phosphatase), CAT, and POX. One of these enzymes, glutathione-S transferase (GST), reduces the effects of lipid peroxidation and hydroperoxides in cells. A number of studies have demonstrated that insect pathogens and toxicants can activate antioxidant enzymes in insects^18,19^.

Humoral reactions have resulted in the production of a diverse range of antimicrobial proteins and phenoloxidase (PO), as well as reactive intermediates of oxygen and nitrogen sources. A variety of mechanisms are employed by entomopathogenic fungi to avoid the defense systems of the insect host^20,21^. Based on experimental observations that insect pathogenic fungi produce several chemical constituents that promote oxidative mechanisms in insects, it was proposed that entomopathogenic fungi inactivate insect antioxidant enzymes^22,23^ . As an insect immune system component, phenoloxidase is vital for wound healing, sclerotization, and melanization. Biological activators convert pro-phenoloxidase (ProPO) to PO, which means the enzyme is completely shutdown. Quinones produced by phenoloxidase can be lethal to intruders^24^.

The entomopathogenic fungi *Metarhizium flavoviride* produce a variety of secondary metabolites that may be explored for use as chemical alternatives in the control of insect pests^25,26^. Among these various metabolites, destruxins, which are cyclic hexadepsipeptides, are the major chemical constituents and almost all are considered toxins. The study species for the current study, *Spodoptera litura* (Fab.) (Lepidoptera: Noctuidae), is a pest of global economic importance and can damage numerous cultivated agricultural crops including, *Gossypium hirsutum*, *Cucumis sativus*, *Cucurbita moschata*, *Momordica charantia*, *Arachis hypogaea*, *Eruca vesicaria*, *Brassica oleracea*, *Brassica oleracea*, and *Brassica oleracea* to name a few^27^.The current study aims to evaluate the effects of infection by entomopathogenic fungus, *M. flavoviride* (Gams and Rozsypal 1956) against the antioxidant and detoxification enzyme defense of *S. litura* under laboratory condition as well as to evaluate the fungi conidia toxicity on a beneficial, bioindicator species, the earthworm *Eudrilus eugeniae*.

## Results

### Larvicidal bioassay

**Figure 1:**
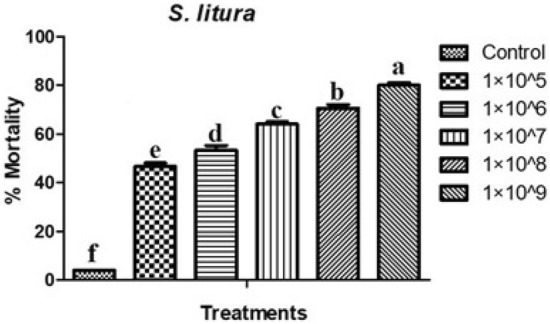
Larvicidal activities of *S. litura* after insect pathogenic fungi spore’s treatment with *M. flavoviride* against *S. litura* larvae. Tukey test (one way ANOVA) indicates that statistical values following the same letter are not significantly different.

*M. flavoviride* fungi conidia were evaluated for larvicidal activity against 3^rd^ instar larvae of *S. litura* with the following test concentrations (1 × 10^5^, 1 × 10^6^, 1 × 10^7^, 1 × 10^8^, and 1 × 10^9^ conidia/mL). Percentage mortality was proportional to the concentration of conidia (Fig. 1). *M. flavoviride* estimated LC_50_ and LC_90_ values against larvae of *S. litura* were 3.0 × 10^5^ and 1.1 × 10^13^ conidia/mL, respectively. Larvae had an 80% mortality rate at the highest concentration (1 × 10^9^ conidia/mL), which was statistically different compared to the control (*F*_(4,10)_ = 15.106; *P* ≤ 0.001). Larval percentage mortality was 80, 70, 64, 53, 46, and 4% at treatment concentrations of 1 × 10^5^, 1 × 10^6^, 1 × 10^7^, 1 × 10^8^, and 1 × 10^9^ conidia/mL and for the control, respectively.

### Total hemocyte count

*S. litura* larvae were exposed to various concentrations of *M. flavoviride* conidia (1 × 10^5^, 1 × 10^6^, 1 × 10^7^, 1 × 10^8^ and 1 × 10^9^ conidia/mL). Hemocyte levels were reduced as fungal spore’s concentration was increased. A higher dose of fungal spores (1 × 10^9^ conidia/mL), caused a 27.73% reduction in haemocyte count after 24 h of treatment and was statistically different compared to the control (*F* (4,10) = 37.976; *P* ≤ 0.000).

. A spore concentration of 1 × 10^5^ conidia/mL caused a small decrease inhaemocyte count (4050 haemocyte/mm^3^) of 2.4%, although this was not statistically different when compared to the control (*F*_(4,8)_ = 6.191; *P* ≤ 0.068).

**Figure 2:**
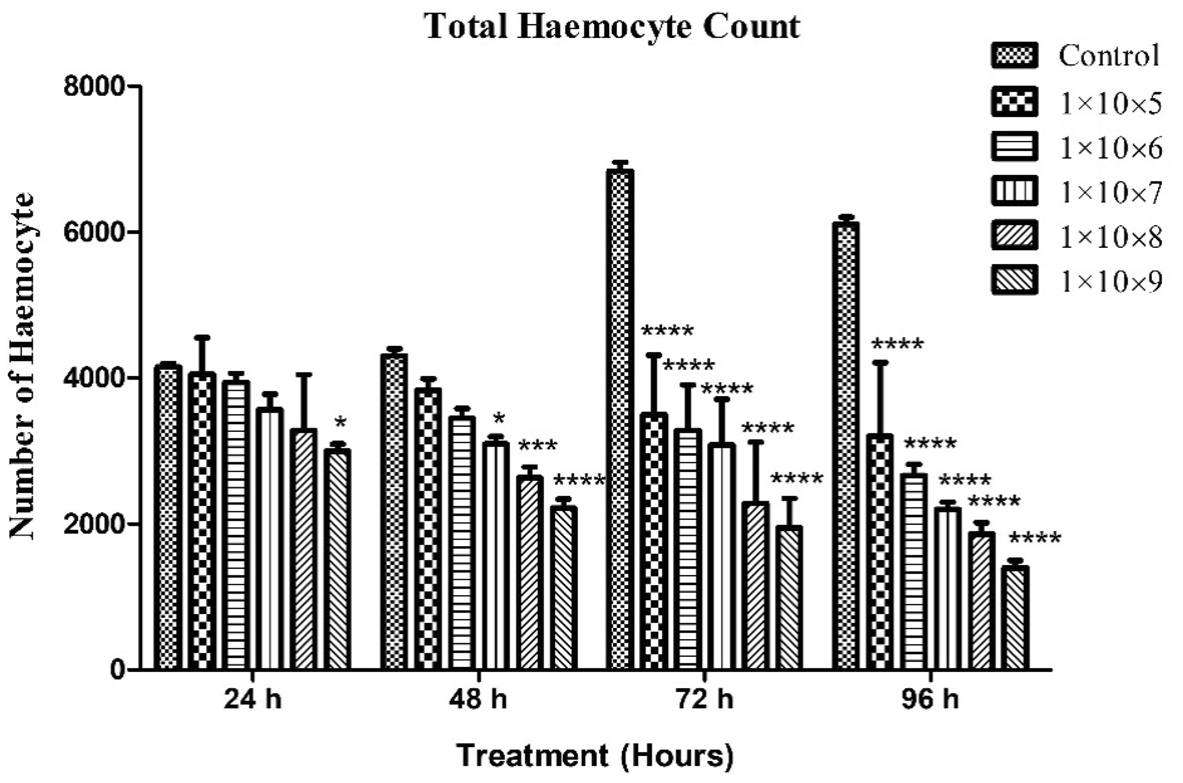
Insect larval haemocyte count after *M. flavoviride* fungi spores’ treatment against *S. litura*. An analysis of variance and multiple comparison test was performed with the data expressed as mean (± S.D) (significantly *p* < 0.05). Compared with control group by ANNOVA. (**** indicates *p* < 0.0001; *** = *p* < 0.001; ** = *p* < 0.01; ** = *p* < 0.05; ns-no significant).

After 48 h, haemocyte count decreased by 10.88, 19.77, 27.91, 38.76, and 48.45% at treatment concentrations of 1 × 10^5^, 1 × 10^6^, 1 × 10^7^, 1 × 10^8^ and 1 × 10^9^ conidia/mL respectively, which proved to be statistically different when compared to the control (*F*_(4,10)_ = 68.505; *P* ≤ 0.000). High-levels of haemocyte reduction occurred in the higher test dose treatment of 1 × 10^9^ conidia/mL (2216 haemocyte/mm^3^) (Fig. 2).

The highest treatment dose of 1 × 10^9^ conidia/mL resulted in a significant reduction in haemocyte count (1950 and 1400 haemocyte/mm^3^) after 72 and 96 h. Haemocyte counts at 72 and 96 h were statistically different from other test concentrations (*F*_(4,10)_ = 142.222; *P* ≤ 0.000); *F*_(4,10)_ = 95.667; *P* ≤ 0.001 respectively).

### Phenoloxidase

**Figure 3:**
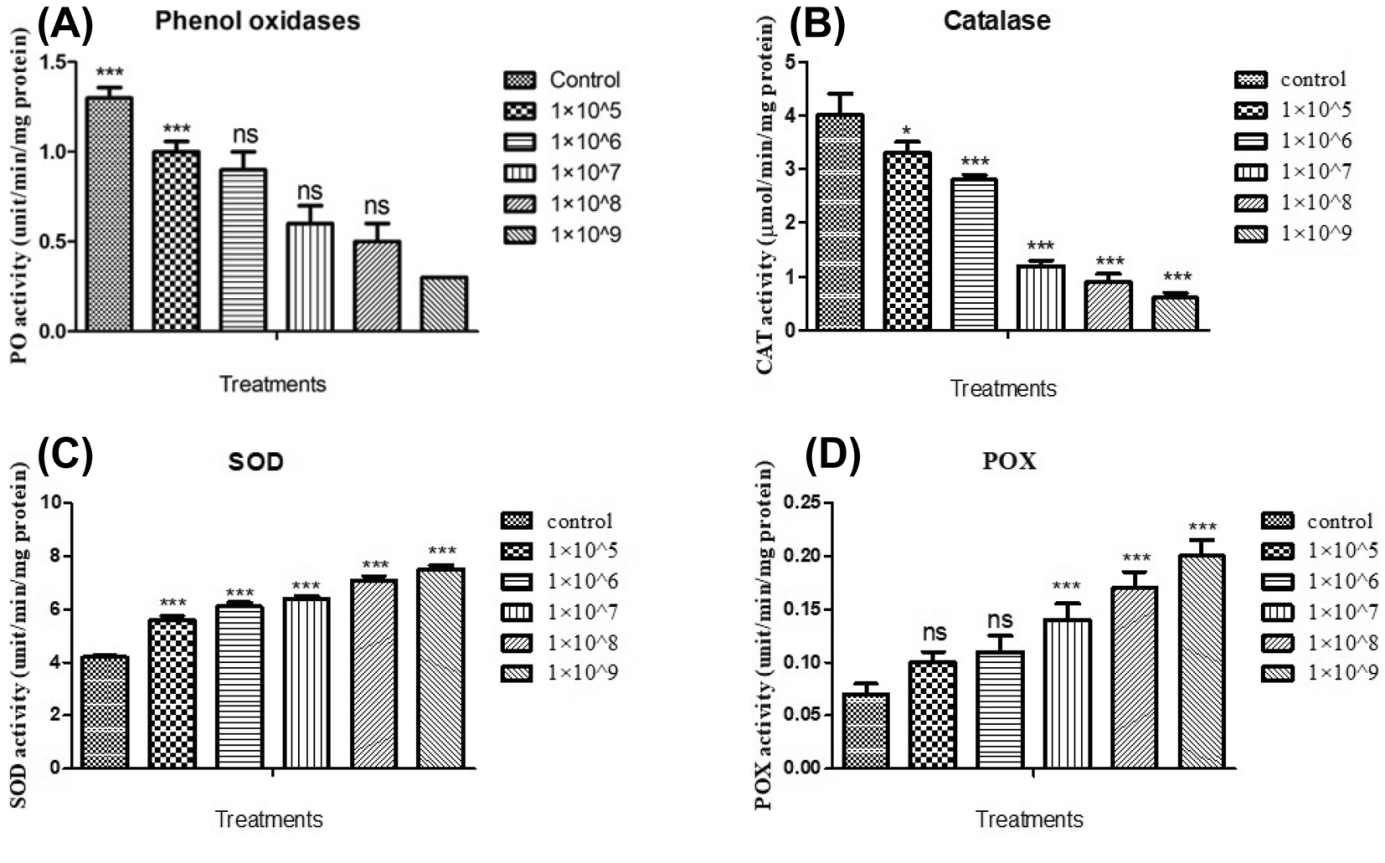
Phenoloxidase activity (**A**), CAT assay (**B**), SOD activity (**C**) and POX activity (**D**) after the treatment with *M. flavoviride* fungi spores against *S. litura*. An analysis of variance and multiple comparison test was performed with the data expressed as mean (± S.D) (significantly p* 0.05). Compared with control group by ANNOVA. (*** indicates *p* < 0.001; ** = *p* < 0.01; * = *p* < 0.05; ns-no significant).

The effect of *M. flavoviride* conidia on phenoloxidase enzyme level was investigated on *S. litura* 3^rd^ instar larvae. Results showed that at the lowest conidial concentration of 1 × 10^5^ conidia/mL, phenoloxidase activity was reduced by24.88%. Conversely, conidia at higher concentrations (1 × 10^9^) showed a decrease in phenoloxidase activity of 65.42% (Fig. 3A). At all treated conidia concentrations, phenoloxidase activity was statistically different compared to the control (*F*_(4,10)_ = 26.893; *P* ≤ 0.001).

### Catalase

Following exposure to *M. flavoviride* conidia, *S. litura* displayed a decrease in catalase activity. After treatment with 1 × 10^5^, 1 × 10^6^, 1 × 10^7^, 1 × 10^8^ and 1 × 10^9^ conidia /mL, catalase activity was reduced by 0.7, 1.2, 2.8, 3.0, and 3.4 mg-1, respectively. Catalase enzyme levels in the control (4 µg-1 protein) significantly differed from the fungi conidia treatment concentrations of 1 × 10^8^ and 1 × 10^9^ conidia/mL (*F*_(4,10)_ = 228.828; *P* ≤ 0.0001), but not the minimal concentration treatment of 1 × 10^5^ conidia/mL (*F*_(4,10)_ = 228.828; *P* ≤ 0.001) (Fig. 3B).

### Superoxide dismutase activity (SOD)

In *S. litura* larvae, SOD enzyme levels were increased when the fungal conidia concentrations were increased. In the treatments involving fungi conidia concentrations of 1 × 10^5^, 1 × 10^6^, 1 × 10^7^, 1 × 10^8^ and 1 × 10^9^ conidia /mL , protein levels of 5.6, 6.1, 6.4, 7.1, and 7.5 Unit/mg/min respectively were obtained. SOD activity in the fungal conidia treatment differed significantly from the control (*F*_(4,10)_ = 81.742; *P* ≤ 0.001) (Fig. 3C).

### Peroxidase activity

After being exposed to *M. flavoviride* conidia, *S. litura* peroxidase activity was increased. After treatment with 1 × 10^9^ conidia/mL, peroxidase activity increased (0.20 units/mg/min protein) and was statistically different (*F*_(4,10)_ = 26.887; *P* ≤ 0.001) when compared to the control (0.0.7 units/mg/min protein). Peroxidase activity, on the other hand, was not significantly affected at lower treatment concentrations of 1 × 10^5^ and 1 × 10^6^ conidia/mL (Fig. 3D).

### Lipid peroxidase activity (LPO)

**Figure 4:**
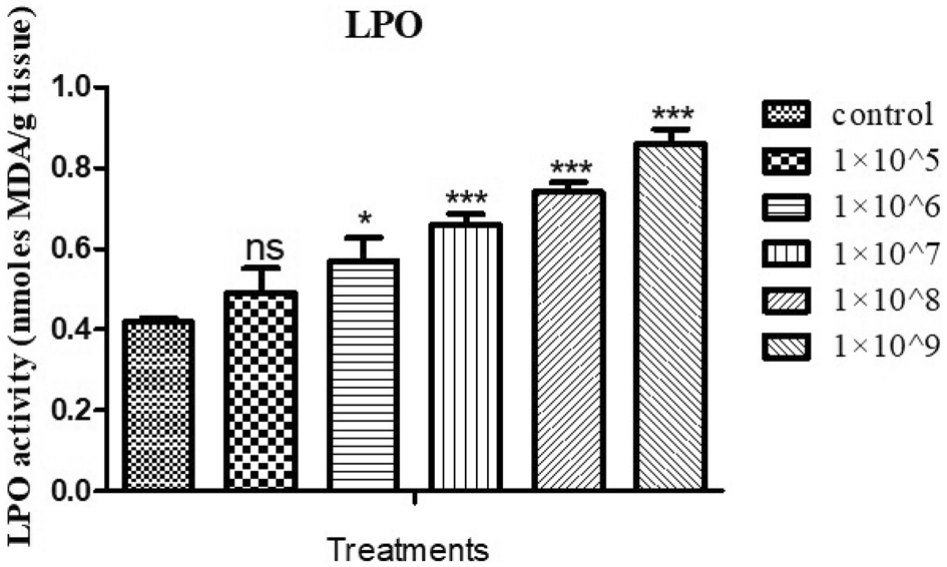
LPO activity in treatment with *M. flavoviride* against *S. litura*. An analysis of variance and multiple comparison test was performed with the data expressed as mean (± S.D) (significantly p* 0.05). Compared with control group by ANNOVA. (*** indicates *p* < 0.001; ** = *p* < 0.01; * = *p* < 0.05; ns-no significant).

*M. flavoviride* fungi conidia can increase the level of LPO enzyme action in a dose-dependent manner. The conidia of *M. flavoviride* exposed to *S. litura* larvae were compared with controls (0.86 Unit/mg/min protein), and showed a rise in LPO enzyme activity of 0.49, 0.57, 0.66, 0.74, and 0.86 Unit/mg/min protein at 1 × 10^5^, 1 × 10^6^, 1 × 10^7^, 1 × 10^8^ and 1 × 10^9^ conidia /mL, respectively (Fig. 4). In the fungal conidia treatment, the LPO enzymatic activity was statistically different compared to the control in treatments containing 1 × 10^5^ conidia/mL (*F*_(4,10)_ = 46.409; *P* ≤ 0.001).

### Acid and alkaline phosphatase assays

**Figure 5:**
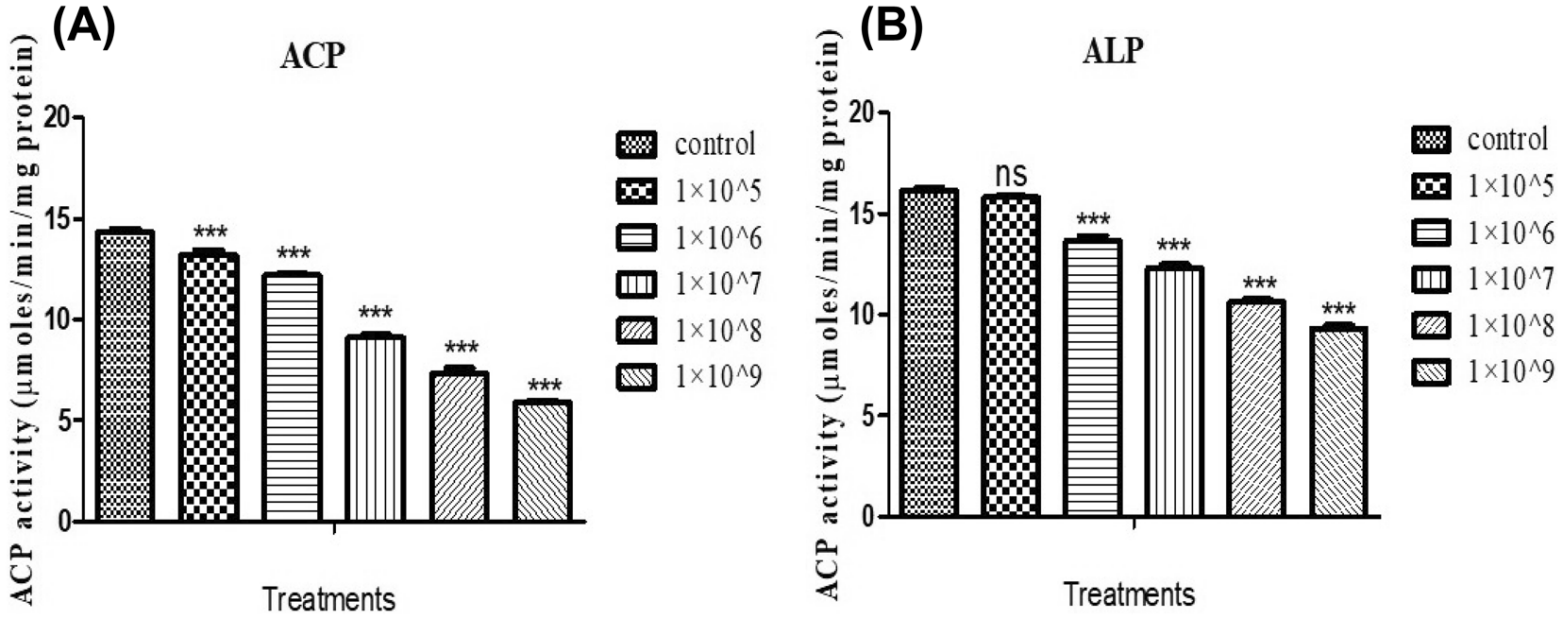
ACP (**A**) and ALP (**B**) activity in treatment with *M. flavoviride* fungi spores against *S. litura*. An analysis of variance and multiple comparison test was performed with the data expressed as mean (± S.D) (significantly p* 0.05). Compared with control group by ANNOVA. (*** indicates *p* < 0.001; ** = *p* < 0.01; * = *p* < 0.05; ns-no significant).

*S. litura* larvae treated with conidia of *M. flavoviride* (1 × 10^5^, 1 × 10^6^, 1 × 10^7^, 1 × 10^8^ and 1 × 10^9^ conidia/mL) demonstrated dose-dependent decreasing activity (13.2, 12.23, 9.13, 7.33, 5.90 Unit/mg/min protein), which was statistically different compared to the control, 14.3 Unit/mg/min protein (*F*_(4,10)_ = 695.214; *P* ≤ 0.001). After treatment with a minimal dose of *M. flavoviride* conidia with minimal dose (1 × 10^5^ conidia/mL), ACP levels were significantly different to the control (*F*_(4,10)_ = 33.0; *P* ≤ 0.005) (Fig. 5A). Overall, *M. flavoviride* conidial treatment was found to increase the levels of hydrolytic enzyme in *S. litura* larvae.

After treatment of *M. flavoviride* conidia, *S. litura* larvae showed significant differences in alkaline phosphatase (ALP) activity. The ALP enzymatic activity was decreased with the treatment of *M flavoviride* conidia (1 × 10^5^, 1 × 10^6^, 1 × 10^7^, 1 × 10^8^ and 1 × 10^9^ conidia/mL) and was significantly different when compared to the control (16.14 Unit/mg/min protein) (*F*_(4,10)_ = 555.25; *P* ≤ 0.000) (Fig. 5B), except for the minimal dose treatment (1 × 10^5^ conidia/mL), which caused a decrease in ALP enzyme activity (15.8 Unit/mg/min protein) (*F*_(1,4)_ = 9.994; *P* ≤ 0.034).

### Toxicity on non-target earthworms

Earthworms were exposed to *M. flavoviride* fungi conidia (1 × 10^5^, 1 × 10^6^, 1 × 10^7^, 1 × 10^8^ and 1 × 10^9^ conidia/mL) and chemical insecticides in filter paper bioassays. Concentrations of 1 × 10^5^, 1 × 10^6^, 1 × 10^7^, 1 × 10^8^ and 1 × 10^9^ conidia/mL, as well as the insecticide cypermethrin (at 100 ppm), were evaluated under laboratory conditions. When compared to *M. flavoviride* conidia at 3 × 10^9^ concentration, cypermethrin was highly toxic. In contrast, *M. flavoviride* fungi conidia were not virulent to earthworms after 96 h of treatment. There were no statistically significant differences between or among the fungi conidia and control groups (*F*_(1, 4)_ = 16.00; *P* = 0.016).

Cypermethrin was found to be the most toxic against earthworms when compared with fungal conidia and the negative control. . In contrast, earthworms treated with fungi conidia, increased the biomass of adult earthworms even after 96 h of exposure. Exposure to 100 ppm cypermethrin (positive control) caused a decreased in earthworm biomass after 24, 48, 72, and 96 h. At 100 ppm concentrations, the greatest weight loss was observed in cypermethrin groups after 24, 48, 72, and 96 h of exposure. The fungal conidia and the control treatment did not show any significant differences (*F*_(1, 4)_ = 4.0; *P* = 0.116).

## Discussion

Insect pathogenic fungi offer effective, cheap, pollution free, and target specific alternatives to synthetic chemicals in the control of insect pests. Worldwide, several entomopathogenic fungi products are widely used in pest control programs against a variety of agricultural pests^18,19,28^, with entomopathogenic fungi control having many advantages when compared to other biocontrol agents^29–32^. Insect pests have evolved many defense systems against insect pathogens and toxins^1,2^. To counter these defenses, insect pathogens have co-evolved with their hosts^33^. Although much is known about the various mechanisms by which fungi overcome their hosts' defenses, the extent to which entomopathogenic fungi impair antioxidative enzymes remains unknown^9^. The present study confirmed *M. flavoviride* fungi to be an effective microbial insecticide against larvae of *S. litura*, with the fungus significantly impairing the immune response of *S. litura*. Results of the larvicidal bioassay indicate that insect pathogenic fungi spores cause dose dependent mortality of *S. litura* larvae. *M. flavoviride* fungi conidia cause harmful effects on the haemocyte levels of *S. litura* larvae, as well as modifications to the antioxidant and detoxification enzyme profiles.

*Metarhizium* species have been shown to have insecticidal potential against a variety of medical and agricultural insect pests, with more than 80–90% mortality rates observed at lower concentration of fungal conidia. Previous research has shown that *M. flavoviride*, *B. bassiana*, *M. anisopliae*, and *A. flavus* can cause significant mortality against insect pests^34–36^. When *M. flavoviride* spores were applied to *S. litura* larvae, the haemocyte counts were decreased significantly in the *M. flavoviride* conidia treated larvae compared to control larvae (fungi conidia free). Insect pathogenic fungi conidia action can reduce protective haemocytes, thus enabling the fungal infection to be successful^36^. In further support of this, it has previously, been shown that exposure of *S. litura* larvae to fungi conidia of *Alternaria alternate* causes a reduction in larval haemocyte levels under laboratory condition^37^.

When faced with microbial infection, PO is the main enzyme that protects insects from^36^, Numerous studies^38–40^ have reported the effects of microbial infection on PO in insects of medical and agricultural importance. For example, *Metarhizium* fungi species have been shown to cause significant mortality in *Schistocerca gregaria* and *Locusta migratoria*, andalso to decrease the levels of protein, phenoloxidase and haemolymph^41^.Furthermore, treatment of insect pathogenic fungi *B. bassiana* (Hypocreales: Clavicipitaceae) conidia resulted in a significant reduction in PO enzyme levels in larvae of *Melanoplus sanguinipes* and *S. exigua*.

In *S. litura, M. flavoviride* fungi conidia infection may be sustained by reduced PO activity. Indeed, after 24 and 48 h of exposure, *M. flavoviride* fungi conidia caused a significant decrease in PO, peroxidase and catalase enzyme activity. Our results showed that catalase and peroxidase enzyme levels changed with treatment of entomopathogenic fungi conidia, but with a significant reduction inlarval PO enzyme level. Catalase (CAT) enzymes, play an important role in the defense against H_2_O_2_ insect pathogens and other toxicants. The current findings demonstrate that *M. flavoviride* fungi conidia pathogenicity increases the CAT enzyme level in *S. litura* larvae. CAT enzymes also play an important role against oxidative stress. Wang et al.^42^ reported that under UV light exposure, CAT enzyme levels are increased in larvae of *S. litura* under laboratory condition. Antioxidant enzymes are suppressed by the excess ROS produced by the crude extract. Extremely high levels of H_2_O_2_ have been shown to prevent SOD enzymes via the development of additional hydroxyl radicals^36–38^. Similarly, the CAT enzyme is sensitive to O_2_ and, at higher concentrations can be inactivated^35–38^.

Our results show that treatment of *M. flavoviride* fungal conidia increased SOD enzyme levels in the larvae of *S. litura* when compared to the control . Similarly, research on insect-fungal interactions indicates that the insect antioxidant enzymes played a significant role in ROS eradication. . Reduced activity of these enzymes reduces insect defense mechanisms^42,43^. Lipid peroxidation (LPO) enzymes areparticularly harmful in insects because, not only are cell membranes comprised of lipids, but lipids also play a vital role in insect reproduction and metamorphosis^42^. Because insect pests are exposed to ROS-inducing agents like insect pathogens or toxicants, insect ROS levels are changed^36,43^. Our findings clearly show that LPO enzymes levels in *M. flavoviride* fungi conidia treated *S. litura* larvae differ compared to controls insects.

The ACP and ALP enzyme systems are involved in insect nutrient passage and food digestion as well as supporting, modulating, and accelerating phagocytosis^36^. Toxicant-containing diets increased the ACP and ALP levels in the hemolymph of the greater wax moth, *Galleria mellonella*, L. (Lepidoptera: Pyralidae). Overall, ACP and ALP activities decreased as heavy metal concentrations increased^44,45^.Our findings are consistent with previous^46^ findings that heavy metal exposure increased enzyme activity levels in the hemolymph of the softshell clam, *Mya arenaria* L. (Myoida: Myidae) . Similarly, *M. flavoviride* significantly reduces ALP and ACP activity levels at low doses (1 × 10^5^ and 1 × 10^6^), but increases ALP and ACP activity at high doses (Fig. 5A, B). According to the present findings, *M. flavoviride* exposure causes a physiological response in *S. litura* that results in increased levels of ALP and ACP enzymes in larvae. Furthermore, insect pathogenic fungi significantly reduced the total count of hemocytes and PO enzyme level after 72 h of treatment. Detoxifying enzyme levels is slightly changed when compared to controls. Results of this study point to *M. flavoviride* fungal infection of *S. litura* larvae by directly affecting insect immune function, with larval immune function being significantly reduced. Larvicidal activity indicates that *M. flavoviride* fungi spores are capable of killing the larvae of *S. litura* within 96 h. This highlights the potential for *M. flavoviride* in the microbial control of *S. litura*.

## Materials and methods

### Insect rearing

We obtained egg masses of *S. litura* from the field at Sanarappatti village, Dharmapuri, Tamil Nadu, India (11.9861°N, 77.9602°E). Eggs were returned to the laboratory and reared under laboratory conditions of 28 ± 1 °C, 80 ± 2% comparative humidity range and 12:12 Light and Dark photoperiod. Larvae were fed on castor leaves (*Ricinus communis*). Once fully developed as adults, adult insects were transferred to an insect cage (15 × 15 × 10 cm) for breeding at a ratio of 2 males: 5 females. A honey + sucrose solution was fed to the adult insects for egg laying, and the cage was protected with cotton fabric. Resultant egg masses were briefly removed and decontaminated by washing with 5% formaldehyde solution for 3 min, then washing with distilled water for 3 min. Decontaminated egg masses were then returned to the cage for hatching.

### Fungal culture

Cultures of fungi were obtained from the Indian type culture collection (ITCC), New Delhi. Entomopathogenic fungi spores were garnered from the culturing medium after 14 days of culture on PDA plates at (27 ± 1 °C) with *M. flavoviride* (ITCC. Acc. No: 6451). To enable separation, the conidia and spores were vortexed for 15 min using sterilized distilled water with 0.1% Tween-80 solution. Conidia concentrations were determined using an improved Neubauer hemocytometer chamber at 40X magnification. To adjust the conidial concentrations, sterile water was used to achieve 1 × 10^5^, 1 × 10^6^, 1 × 10^7^, 1 × 10^8^, 1 × 10^9^ conidia/mL.

### Fungal bioassay

An insect dip method was used to induce fungal infection. In the fungal conidia aqueous suspension, each third instar *S. litura* larva was dipped for 3 s using a concentration of 1 × 10^5^, 1 × 10^6^, 1 × 10^7^, 1 × 10^8^, or 1 × 10^9^ conidia/mL. Following conidia treatment, the larvae were kept in Petri plates at a density of 25 larvae per dish. The negative control (conidia-free) insects were exposed to distilled water in place of the conidia suspension. Castor leaf was used as a food source in a bioassay container after larval treatment. Larval mortality was recorded at 24, 48, 72, and 96 h post treatment and compared to the control group. Dead insect larvae were separated from the bioassay container and cleaned with 70% ethanol for 15 min and then cleaned twice with sterilized distilled water. The larvicidal activity assay was performed in triplicate and % mortality was calculated every 24 h after treatment. Percentage mortality was calculated using the formula (1) and the corrected percentage mortality using Abbott’s formula^47^(2).

1 $$ {\text{Percentage }}\;{\text{of}}\;{\text{ mortality}} = \frac{{{\text{Number}}\;{\text{ of }}\;{\text{dead }}\;{\text{larvae}}}}{{{\text{Number}}\;{\text{ of}}\;{\text{ larvae }}\;{\text{introduced}}}} \times 100 $$

2 $$ {\text{Corrected}}\;{\text{ percentage }}\;{\text{of}}\;{\text{ mortality = }}\left( {\frac{{{1 - }n \, \;{\text{in}}\;{\text{ T }}\;{\text{after}}\;{\text{ treatment}}}}{{n\;{\text{ in }}\;{\text{C}}\;{\text{ after}}\;{\text{ treatment}}}}} \right) \times 100 $$

Where, T—the number of larvae in treated groups; C—the number of larvae in control groups.

### Hemolymph collection

Each individual larva was weighed at each of the subsequent time intervals: 24 h, 48 h, 72 h and 96 h. Following weighing, hemolymph was extracted from the insect by piercing the last proleg with a tiny needle of a Hamilton syringe. Using three 1.5 ml tubes, each sample of hemolymph was divided equally. A hemocyte count was conducted using hemolymph mixed with EDTA anticoagulant in phosphate saline (PBS, pH 7.4 ± 1) with glycerol. 8 µl of hemolymph was added to 400 µl of PBS for the ProPO and protein assays. Following this, all samples were frozen at a temperature of − 20 °C.

### Total Hemocyte count (THC)

A blood cell pipette was accustomed to draw up hemolymph to the 0.5 mL mark, then diluted with Tauber-Yeager fluid (NaCl = 4.65 g, KCl = 0.15 g, CaCl_2_ = 0.11 g, Gentian violet = 0.005 g, Acetic acid = 0.125 ml, Distilled water = 100 ml)^21^. the pipette was then mixed gently for few minutes. The hemocytes were counted under a light microscope (Olympus) at 40X and counted according to Jones' formula^48^.

$$ {\text{Number}}\;{\text{ of}}\;{\text{ hemocytes}}/{\text{mm}}^{{3}} = \frac{{{\text{X }} \times {\text{ dilution }} \times { 1}0 \, \times { 1}00}}{{{\text{Number}}\;{\text{ of}}\;{\text{ smallest}}\;{\text{ squares }}\;{\text{counted}}}} $$

### Phenoloxidase activities

Based on Cotter and Wilson’s method^49^, phenoloxidase activity was calculated. A solution of 400 µl of ice-cold phosphate buffered saline (PBS, pH 7.4) was mixed with 8 µl of hemolymph. Samples were frozen at -20 °C until they were thawed for subsequent measurement. 100 µl of 20 mM L-Dopa was incubated with 100 µl of the hemolymph/PBS solution. A spectrophotometer was used to measure the absorbance at 475 nm after 30 min. An enzyme unit is equal to the amount of enzyme necessary to increase the absorbance of a sample by 0.001 every minute. The amount of PO in the sample was calculated in PO units.

### Homogenate preparation

A 0.1 M phosphate buffer (pH 7.2) comprised of 1 mM EDTA, 1 mM DTT, 1 mM PTU, 1 mM PMSF and 20% glycerol was used to homogenize ten larvae treated with *M. flavoviride*. Ice was used to chill the insects before blending them. The homogenate from the homogenized tissues was centrifuged for 15 min at 4 °C and 10,000 g to remove solid debris and cellular components. In addition to measuring Acid phosphatase, Alkaline phosphatase, Lipid peroxidase, and Superoxide dismutase, the supernatant was transferred into a new tube and directly placed on ice for CAT, POX, SOD, and ALP enzymatic analysis. The total insect protein content was evaluated^50^.

### Catalase assay

The decomposition rate of H_2_O_2_ by catalase enzyme was used to calculate CAT activity^51^. CAT enzyme activity was calculated in µmoles of H_2_O_2_ decomposition/min/mg protein.

### Superoxide dismutase assay (SOD)

SOD enzymatic activity was calculated as previously detailed by^52^. In 3 ml glass spectrophotometer cuvettes, reaction mixtures were arranged by adding 2.8 ml of Tris–EDTA (50 mM Tris and 10 mM EDTA, pH 8.2) buffer and 50 µl of enzyme mixture. The contents were united, and the final volume was concentrated to 2.9 ml using a Tris–EDTA solution. The reaction in the cuvette was started by adding 100 µl of Pyrogallol (15 mM). The rates of auto-oxidation were observed in the UV-Visible spectrophotometer (Systronics) at 440 nm for 3 min, and absorbance range was calculated. One-unit total SOD enzymatic activity was evaluated as the amount of protein per milligram that inhibited pyrogallol autoxidation by 50%. SOD activity was measured in units per milligram of protein.

### Peroxidase assay

POX enzymatic activity was calculated using the method detailed by Reddy et al.^53^ by using a UV–Vis spectrophotometer at the wavelength 430 nm. POX enzymatic activity was calculated in mol min^-1^ mg^-1^ protein.

### Lipid peroxidation assay

Ohkawa et al.^54^ used this method to calculate the lipid peroxidase activity. Malondialdehyde is formed as a result of the lipid peroxidation process (MDA). Thiobarbituric acid substances are designed as a byproduct of lipid peroxidation and can be detected using the TBARS assay, which used thiobarbituric acid as a reagent. A 0.1-ml sample of the enzyme solution was collected, and 1.9 ml of 0.1 M sodium phosphate buffer at pH was added to it. The mixture was then incubated for one hour at 37 °C. This solution was precipitated with 10% TCA and then centrifuged at 6000 rpm for 10 min before the supernatant was collected. 1 µ of 1% TBA was subsequently added to the supernatant. For 15 min, the sample was boiled in a water bath. After boiling, the supernatant was cooled and the absorbance at 532 nm was measured. MDA was measured in nanomoles per hour per milligram of protein.

### Acid and alkaline phosphatase assays

The enzymatic levels of acid and alkaline phosphatases in larvae homogenates were calculated^55^. Acid phosphatase activity was determined by combining 50 µl of larval homogenate with 450 µl of 50 mM sodium acetate buffer at optimal pH of 4.6 or pH 4.0. To determine alkaline phosphatase activity, 20 µl of larval homogenate was mixed with an equal volume of the respective buffer containing 12.5 mM p-nitrophenyl phosphate and diluted to 500 µl with 50 mM Tris–HCl buffer at the optimal pH of 8.0. After a 15 min incubation period in a water bath at 37 °C, the enzymatic reaction was immobilized by addition of 100 µl of 0.5 N NaOH buffer and centrifuging at 3500 rpm for 10 min. At 440 nm, the absorbance of the resulting clear supernatants was measured.

### Earthworm rearing

Under laboratory conditions, *E. eugeniae* was maintained at room temperature (27 ± 2 °C) on crop residues fed with cattle dung. Hand sorting was used to separate earthworms from the soil for experiments. Earthworms were washed in tap water to remove soil particles from their bodies and weighed. Separated earthworm cocoons were counted and then placed in isolated bedding.

### Contact toxicity assays

The contact toxicity assay was performed on filter paper in accordance with Kühnel^56^. Five concentrations of entomopathogenic fungi spores, cypermethrin (positive control), and a negative control were individually mixed onto small pieces of filter paper and transferred into petri-plates, each with three earthworms. Three replicates of each concentration were carried out. Treated earthworms were placed in plates in the dark at 27 ± 2 °C and 85–90% R.H. for 96 h, before mortality rates were calculated.

### Artificial soil toxicity analysis

Kühnel’s^56^ procedure was followed for the artificial soil toxicity bioassay. The artificial soil was comprised of 12% crushed sphagnum peat, 19% kaolinite clay, and 69% tiny sand. For each test concentration, the anticipated amount of chemical insecticide (cypermethrin 100 ppm) and various fungi concentrations of fungal spores were mixed into a small amount of fine sand. Each 500 mL glass jar was filled with 0.65 kg of soil, and 15 mature earth worms were introduced. Polypropylene caps were used to loosely cover the container to allow air circulation. Containers were maintained at 27 ± 2 °C with 85–90% relative humidity under constant light. Earthworm mortality was determined 96 h after treatment.

### Data analysis

All of the insect larval enzymatic assays defined above were made with five concentrations, each with three replications. Using the PRISM, Version-6 software, the data from enzyme assays were subjected to analysis of variance, followed by Dunnett's multiple comparison test (Graph Pad Software Inc, USA). *p*-values of 0.05 were measured as statistically significant.

## Data Availability

During the present research entities, the datasets gathered and generated from the analysis after entomopathogenic fungi spore treatment and, the evaluated biological results are available from the corresponding author on reasonable request.

## References

[1] Marrone PG (2019). Pesticidal natural products–Status and future potential. Pest Manag. Sci.

[2] Chandler D (2008). Microbial biopesticides for integrated crop management: an assessment of environmental and regulatory sustainability. Trends. Food Sci. Technol.

[3] Khater HF (2012). Prospects of botanical biopesticides in insect pest management. Pharmacol.

[4] Grant, W.P. *et al. Biopesticides: Pest management and regulation*. CABI. (2010).

[5] Lacey LA (2015). Insect pathogens as biological control agents: Back to the future. J. Invert. Pathol.

[6] Lacey LA, Shapiro-Ilan DI (2008). Microbial control of insect pests in temperate orchard systems: Potential for incorporation into IPM. Annu. Rev. Entomol..

[7] Kachhawa D (2017). Microorganisms as a biopesticides. J. Entomol. Zool. Stud..

[8] Singh, A., Bhardwaj, R. & Singh, I.K. Biocontrol agents: potential of biopesticides for integrated pest management. In Biofertilizers for sustainable agriculture and environment Springer, Cham 413–433 (2019).

[9] Jayanthi PK (2015). *Aspergillus flavus* impairs antioxidative enzymes of *Sternochetus mangiferae* during mycosis. J. Invert. Pathol..

[10] Poinar GO, Thomas GM (2012). Laboratory guide to insect pathogens and parasites.

[11] Maina UM (2018). A review on the use of entomopathogenic fungi in the management of insect pests of field crops. J. Entomol. Zool. Stud.

[12] Mannino MC (2019). Is the insect cuticle the only entry gate for fungal infection? Insights into alternative modes of action of entomopathogenic fungi. J. Fungi.

[13] Rosales, C. and Vonnie, S., 2017. Cellular and molecular mechanisms of insect immunity. *Insect Physiol Ecol* 179–212 (2017).

[14] Tsakas S, Marmaras VJ (2010). Insect immunity and its signalling: An overview. Invert Survival J.

[15] Lavine MD, Strand MR (2002). Insect hemocytes and their role in immunity. Insect Biochem. Mol Biol.

[16] Amaral IMR (2010). Circulating hemocytes from larvae of *Melipona scutellaris* (Hymenoptera, Apidae, Meliponini): Cell types and their role in phagocytosis. Micron.

[17] Kamata H, Hirata H (1999). Redox regulation of cellular signaling. Cell Signal.

[18] Vivekanandhan, P. Swathy, K. & Shivakumar, M.S. Stability of insecticidal molecule aucubin and their toxicity on *Anopheles stephensi*, *Aedes aegypti*, *Culex quinquefasciatus* and *Artemia salina*. *Int. J. Trop. Insect Sci* 1–15 (2022).

[19] Vivekanandhan P (2018). Toxicity of *Beauveria bassiana*-28 mycelial extracts on larvae of *Culex quinquefasciatus* mosquito (Diptera: Culicidae). Int. J. Env. Res. Pub Health.

[20] Ortiz-Urquiza A, Keyhani NO (2013). Action on the surface: Entomopathogenic fungi versus the insect cuticle. Insects.

[21] Lu HL, Leger RS (2016). Insect immunity to entomopathogenic fungi. Adv. Genet.

[22] Rasool, S. Entomopathogenic fungal endophytes in plant-fungus herbivore interactions: Exploring the importance of selected physiological responses in regulation of arthropod populations. (2020).

[23] Wang Y, Casadevall A (1994). Susceptibility of melanized and nonmelanized *Cryptococcus neoformans* to nitrogen-and oxygen-derived oxidants. Inf. Imm.

[24] Au, C.P.Y. Haemolytic virulence factors in photorhabdus luminescens strain w14. University of Bath (United Kingdom). (2004).

[25] Vivekanandhan P (2022). Insecticidal efficacy of *Metarhizium anisopliae* derived chemical constituents against disease-vector mosquitoes. J. Fungi.

[26] Vivekanandhan P (2020). Larvicidal toxicity of *Metarhizium anisopliae* metabolites against three mosquito species and non-targeting organisms. PLoS ONE.

[27] Marrone PG (2019). Pesticidal natural products–status and future potential. Pest Manag. Sci..

[28] Vivekanandhan P (2021). Insecticidal efficacy of microbial-mediated synthesized copper nano-pesticide against insect pests and non-target organisms. Int J. Env. Res. Pub. Health.

[29] Starnes RL, Liu CL, Marrone PG (1993). History, use, and future of microbial insecticides. Am. Entomol..

[30] Skinner, M., Parker, B.L. & Kim, J.S. Role of entomopathogenic fungi in integrated pest management. *Integ Pest Manag*169–191 (2014).

[31] Islam W (2021). Insect-fungal-interactions: A detailed review on entomopathogenic fungi pathogenicity to combat insect pests. Microb. Pathog.

[32] Shah PA, Pell JK (2003). Entomopathogenic fungi as biological control agents. Appl. Microbiol. Biotechnol..

[33] Walling LL (2009). Adaptive defense responses to pathogens and insects. Advan. Bot. Res.

[34] Borisade OA, Magan N (2014). Growth and sporulation of entomopathogenic *Beauveria bassiana*, *Metarhizium anisopliae*, *Isaria farinosa* and *Isaria fumosorosea* strains in relation to water activity and temperature interactions. Biocon. Sci. Technol.

[35] Li, M. *et al.* Selection of *Beauveria* isolates pathogenic to adults of *Nilaparvata lugens*. *J. Insect Sci* **14** (2014). 10.1093/jis/14.1.32 PMC4206227 25373179

[36] Karthi S (2018). Effect of Aspergillus flavus on the mortality and activity of antioxidant enzymes of Spodoptera litura Fab (Lepidoptera: Noctuidae) larvae. Pest. Biochem. Physiol..

[37] Kaur HP (2015). Studies on immunomodulatory effect of endophytic fungus *Alternaria alternata* on *Spodoptera litura*. J. Asia-Pacific Entomol.

[38] González-Santoyo I, Córdoba-Aguilar A (2012). Phenoloxidase: A key component of the insect immune system. Entomol. Exper. Appl.

[39] Dubovskiy IM (2011). The effects of dietary nickel on the detoxification enzymes, innate immunity and resistance to the fungus *Beauveria bassiana* in the larvae of the greater wax moth *Galleria mellonella*. Chemos..

[40] Binggeli O, Neyen C, Poidevin M, Lemaitre B (2014). Prophenoloxidase activation is required for survival to microbial infections in Drosophila. PLoS Pathog..

[41] Rajendran S, Vasudevan S (2020). Activation of prophenoloxidase and hyperglycemia as indicators of microbial stress in the blue swimmer crab Portunus pelagicus. Mar. Pollut. Bull..

[42] Wang YT, Yang CH, Huang KS, Shaw JF (2021). Chlorophyllides: Preparation, purification, and application. Biomolecules.

[43] Duarte, J.P. Impacto de diferentes estresses no peso e estado imunológico em Periplaneta americana (Linnaeus, 1758)(Blattaria, Blattidae).

[44] Wu G, Yi Y (2015). Effects of dietary heavy metals on the immune and antioxidant systems of *Galleria mellonella* larvae. Comp. Biochem. Physiol. Part C Toxicol Pharmacol..

[45] Qin Q (2012). Immune responses and ultrastructural changes of hemocytes in freshwater crab *Sinopotamon henanense* exposed to elevated cadmium. Aquat. Toxicol..

[46] Rodrick GE (1979). Selected enzyme activities in* Mya arenaria* hemolymph. Comp. Biochem. Physiol. B, Comp. Biochem..

[47] Abbott WS (1925). A method of computing the effectiveness of an insecticide. J. Econ. Entomol.

[48] Jones JC (1962). Current concepts concerning insect hemocytes. Am. Zool.

[49] Cotter SC, Wilson K (2002). Heritability of immune response function in the caterpillar *Spodoptera littoralis*. Heredity.

[50] Lowry OH (1951). Protein measurement With the Folin-Phenol reagent. J. Biol. Chem.

[51] Luck, H. Catalase. In: Bergmeyer HU, editor. Methods of enzymatic analysis New York. Academic Press. 885–893 (1971).

[52] Marklund SL, Marklund G (1974). Involvement of the superoxide anion radical in the autoxidation of pyrogallol and a convenient assay for superoxide dismutase. Eur. J. Biochem..

[53] Reddy KP (1995). Effect of light and benzyladenine on dark treated graving rice (*Oryza sativa*) leaves - changes in peroxidase activity. Plant Cell Physiol..

[54] Ohkawa H (1979). Assay for lipid peroxides in animal tissues by thiobarbituric acid reaction. Ann. Biochem..

[55] Asakura K (1978). Phosphatase activity in the larva of the euryhaline mosquito, *Aedes togoi* Theobold, with special reference to sea water adaptation. J. Exp. Mar. Biol. Ecol.

[56] Kühnel D, Nickel C (2014). The OECD expert meeting on ecotoxicology and environmental fate—Towards the development of improved OECD guidelines for the testing of nanomaterials. Sci. Total Environ..

